# Molecular Mechanisms Underlying *Mimosa acutistipula* Success in Amazonian Rehabilitating Minelands

**DOI:** 10.3390/ijerph192114441

**Published:** 2022-11-04

**Authors:** Sidney Vasconcelos do Nascimento, Héctor Herrera, Paulo Henrique de Oliveira Costa, Felipe Costa Trindade, Isa Rebecca Chagas da Costa, Cecílio Frois Caldeira, Markus Gastauer, Silvio Junio Ramos, Guilherme Oliveira, Rafael Borges da Silva Valadares

**Affiliations:** 1 Instituto Tecnologico Vale, Rua Boaventura da Silva 955, Belém 66050-090, PA, Brazil; 2 Programa de Pos-Graduacão em Genética e Biologia Molecular, Universidade Federal do Pará, Belém 66075-110, PA, Brazil; 3 Laboratorio de Silvicultura, Departamento de Ciencias Forestales, Facultad de Ciencias Agropecuarias y Medioambiente, Universidad de La Frontera, Temuco 4811230, Chile

**Keywords:** abiotic stress, Amazon, *Canga*, iron mining, mineland rehabilitation, proteomics, symbiosis

## Abstract

*Mimosa acutistipula* is endemic to Brazil and grows in ferruginous outcrops (*canga*) in Serra dos Carajás, eastern Amazon, where one of the largest iron ore deposits in the world is located. Plants that develop in these ecosystems are subject to severe environmental conditions and must have adaptive mechanisms to grow and thrive in *cangas*. *Mimosa acutistipula* is a native species used to restore biodiversity in post-mining areas in *canga*. Understanding the molecular mechanisms involved in the adaptation of *M. acutistipula* in *canga* is essential to deduce the ability of native species to adapt to possible stressors in rehabilitating minelands over time. In this study, the root proteomic profiles of *M. acutistipula* grown in a native *canga* ecosystem and rehabilitating minelands were compared to identify essential proteins involved in the adaptation of this species in its native environment and that should enable its establishment in rehabilitating minelands. The results showed differentially abundant proteins, where 436 proteins with significant values (*p* < 0.05) and fold change ≥ 2 were more abundant in *canga* and 145 in roots from the rehabilitating minelands. Among them, a representative amount and diversity of proteins were related to responses to water deficit, heat, and responses to metal ions. Other identified proteins are involved in biocontrol activity against phytopathogens and symbiosis. This research provides insights into proteins involved in *M. acutistipula* responses to environmental stimuli, suggesting critical mechanisms to support the establishment of native *canga* plants in rehabilitating minelands over time.

## 1. Introduction

Iron mining in the eastern Amazon occurs in one of the world’s most significant biodiversity hotspots, where the Carajás Mineral Province is located [1]. The iron ore deposits in the Carajás Mineral Province are found in ferriferous savannas, known as *canga* [1,2]. *Canga* environments are considered severe due to high temperatures, strong winds, and soils characterized by low availability of essential nutrients, especially phosphorus, acidic pH (pH~4), low water retention capacity, and high levels of heavy metals such as iron [2,3,4]. These factors impair plant adaptations, providing a selection of plant species adapted to establish themselves in these environments [5].

Iron ore extraction occurs mainly in open-cast mines, transforming landscapes and altering soil’s physical, chemical, and biological properties [6,7,8,9]. Soils in open pits after mining are poor in nutrients and organic matter, showing resistance to root penetration and plant growth in compaction and drying out [10,11]. These changes underline the loss of specific ecological services, affecting the biodiversity and sustainability of native areas [12]. In this context, mineland rehabilitation is necessary to reduce net biodiversity loss and reestablish the ecosystem characteristics [13]. The steps that contribute to the recovery of areas impacted by iron mining include restoring the physical and chemical properties of the soil, selecting native species, controlling invasive species, and monitoring the success of rehabilitation [6,14,15,16,17]. Thus, screening native plants capable of adapting to these harsh environments represents a fundamental step [2,3,18,19]. These species must have a facilitating role, ease of propagation, importance in the composition of native canga communities, and ease of growth in the rehabilitating minelands [10]. Such species represent a potential for the revegetation of mined areas, contributing to the reduction of the loss of local biodiversity and the recovery of ecosystem services [15].

Plants from the Fabaceae family have been described as one of the pioneer species to be used in rehabilitation programs in the eastern Amazon [14,16,19,20]. Among them, *Mimosa acustitipula* var *ferrea* Barneby (Fabaceae) has been classified as one of the native plants with high performance in rehabilitating minelands [14,16,17]. Nutrient use efficiency and non-specific interactions with soil microorganisms have been revealed as principal mechanisms underlying the establishment of this species in post-mining areas [16,21]. Modifications in plant metabolism are common characteristics of plants growing in minelands [22,23]. However, little is known about the adaptive molecular mechanisms developed by this species throughout its evolutionary history.

In recent years, proteomics has become a powerful tool for studying environmental processes, including abiotic stress tolerance, plant diseases, phytohormone metabolism, and growth promotion [24,25,26]. Recent studies have demonstrated that proteomics can be a valuable tool to explore the metabolisms of plants and microorganisms growing in severe ecosystems, including their capabilities to improve ecosystem-related services in rehabilitating minelands [20,27]. We hypothesized that *M. acutistipula* develops mechanisms of stress tolerance in the native *canga,* which allow its establishment in rehabilitating minelands. Analyzing protein profiles makes it possible to identify critical proteins involved in responses to environmental stresses. The adaptation capacities of these species can be deduced in the face of potential stressors in rehabilitating minelands. Identifying these modifications is essential to understanding the critical process underlining the adaptation of native plants to rehabilitating minelands. Such information reinforces the importance of species such as *M. acutistipula* in the rehabilitation of minelands and their contribution to reducing the loss of biodiversity in the Amazon. This is the first study that aimed to identify critical proteins in the responses to environmental stresses in the roots of *M. acutistipula* grown in *canga,* which may support the establishment of this species in rehabilitating minelands.

## 2. Materials and Methods

### 2.1. Substrate Sampling and Analyses

Four soil samples were collected near *M. acutistipula* roots naturally growing in *canga* and in an RM project at a depth of 20 cm. After being air-dried, the samples were sieved using a 2 mm mesh. Variables were evaluated considering the fundamental characteristics of these environments, such as P and Fe contents, organic matter, organic carbon, pH, and soil granulometry. The pH was determined in a 1:2.5 soil substrate-to-water ratio. The organic carbon content was determined using the potassium dichromate (K_2_Cr_2_O_7_) method. The available P and Fe were determined using the Mehlich-1 method (0.05 mol L^−1^ HCl + 0.0125 mol L^−1^ H_2_SO_4_), S-SO_4_^−2^ by calcium phosphate monobasic at 0.01 M. The soil texture was determined as described by [28].

### 2.2. Root Sampling

Composite root samples from four plants of the *M. acutistipula* species were sampled from each environment. The samples were obtained in a native shrub *canga* (60°00′41.0″ S 50°17′45.0″ W) and in waste piles of a rehabilitating mineland (60°20′32.0″ S 50°07′04.0″ W) in Serra dos Carajas, Pará state, northern Brazil, at the end of the wet season (May 2018). The region’s climate is tropical warm, with a rainy season from November to March, a dry season from May to September, an average annual rainfall of 2033 mm, and temperatures varying between 25 °C and 26 °C [29,30]. The rehabilitation program of the mining area started in 2014, where native seeds were dispersed with hydroseeding containing NPK fertilization (04-14-08). Five grams of secondary roots (depth of 5 to 30 cm) from four individuals was collected, kept in a cold phenol/SDS buffer, and transported to the laboratory for further processing.

### 2.3. Protein Isolation

The roots of each plant were pooled and submitted to a standard protein extraction protocol from plant tissues, according to Wang et al. [31], with the modifications of Nascimento et al. [32]. The roots of the four selected plants (300 mg each) were macerated in liquid nitrogen using a mortar and a pestle. Then, 10 mL of a buffer containing 1.5 M sucrose, 1.5 M Tris-Hydrochloride pH 8, 10% sodium dodecyl sulfate (SDS), 100 mM phenylmethylsulfonyl fluoride (PMSF), polyvinylpolypyrrolidone (PVPP), ultrapure water with the addition of 100 μL of protease inhibitor (Sigma-Aldrich, St. Louis, MO, USA) and 500 μL of β-mercaptoethanol was added to each sample. After that, the samples were sonicated five times for 30 s at room temperature. The extracts were divided into 10 microtubes, and 700 μL of phenol was added per microtube. The samples were vortexed for 15 min and centrifuged for 8 min at 14,000 rpm to allow the phenolic phase separation. Afterward, the phenolic phase was transferred to a new microtube and repeated to eliminate any aqueous phase or SDS residue. About 1.3 mL of 100 mM ammonium acetate was added to each microtube, and the proteins were precipitated for 24 h at −80 °C. The samples were centrifuged at 14,000 rpm for 8 min, and the supernatants were discarded. The precipitates were transferred to new microtubes and washed with 80% acetone four times. The last washing step was made with 70% ethanol, and the precipitates were dried at room temperature in a vacuum concentrator for 7 min. Finally, the extracts were solubilized in 200 μL of 0.2% RapiGest (Waters, Milford, MA, USA) and stored for further analysis.

### 2.4. Protein Identification and Data Analysis

Five micrograms of the peptides were analyzed in a NanoACQUITY UPLC ultra-performance liquid chromatography (Waters, Milford, MA, USA), configured for fractionation in two dimensions as reported in Herrera, et al. [33] and five analytical replicates. The first dimension used a 5 μm XBridge BEH130 C18 (300 μm × 50 mm) (Waters, Milford, MA, USA) and a Symmetry C18 5 μm (180 μm × 20 mm) trapping column at a flow rate of 2000 μL min^−1^. The second dimension used a 1.7 μm BEH130 C18 1.8 μm (100 μm × 100 mm) analytical column at a flow rate of 400 μL min^−1^. The samples were separated into five fractions with a gradient of 10.8, 14.0, 16.7, 20.4, and 65.0% acetonitrile. The chromatograph was coupled to a NanoLock ESI-Q-ToF SYNAPT G2-S (Waters) mass spectrometer. The acquisition ranged from 50 to 2000 Da, in MS^E^ mode (data-independent analysis) at a scan rate of 0.5 s and an interscan delay of 0.1 s. For each sample, two replicates were obtained.

The data were processed using the Progenesis QI software (Waters) for identification and quantification, using the Viridiplantae database from UniProt (UniProtKB/swiss-prot, uniprot.org). Protein identification was accepted if the probability of identifying peptides was greater than 90% and proteins with 95%. The significance levels of the differentially abundant proteins were determined by applying the ANOVA test (*p*-value < 0.05) performs by Progenesis QI. To verify the influences of the environments on the sets of differentially abundant proteins with *p*-value < 0.05 and fold change (FC) ≥ 2 in the samples, a principal component analysis (PCA) was performed using the R software v3.6.3 (R Core Team 2018; https://www.R-project.org (accessed on December 2021), with the packages FactoMineR, Factoshiny and Factoextra. Gene Ontology analyses of differentially abundant proteins were performed using the OmicsBox v1.2.4 (bioBam) and Uniprot (UniProtKB/swiss-prot, uniprot.org (accessed on January 2022). Heatmaps of differentially abundant proteins were developed using R software v.3.6.3 (package pheatmap v.1.0.12, ggplot2 v.3.3.5, colorspace 2.0-2, and grid 4.0.4).

## 3. Results

### 3.1. Substrate Properties

Table 1 shows some of the determinant characteristics in the adaptation of plants in the *canga* or RM environments. *Canga* soils showed higher organic matter and carbon content, probably due to greater biodiversity in this environment. As expected, the soils had a more acidic pH in these environments, which reflects soil correction in RM environments before revegetation. The high Fe content in native *canga* soils can also be observed. This result was expected due to the revegetation in RM being carried out on waste piles obtained after mining. Finally, RM soils had higher sand and silt content, while *canga* soils had higher clay content. Such physical characteristics determine permeability and water retention capacity, among other factors in these soils.

### 3.2. Protein Profiles

**Figure 1:**
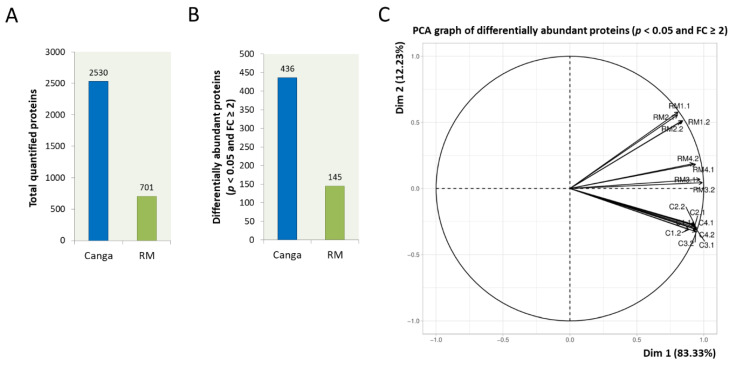
Differentially abundant proteins and PCA from the proteomes of *M. acutistipula* roots sampled in a rehabilitating mineland (RM) and a native shrub *canga*. (**A**) Total quantified proteins in roots from each environment. (**B**) Number of most abundant proteins in roots from each environment considering a *p*-value < 0.05 and fold change ≥ 2. (**C**) PCA of differentially abundant proteins with *p*-value < 0.05 and fold change ≥ 2 comparing replicates of roots from RM (RM1.1, RM1.2, RM2.1, RM2.2, RM3.1, RM3.2, RM4.1, and RM4.2) and *canga* (C1.1, C1.2, C2.1, C2.2, C3.1, C3.2, C4.1, and C4.2). The proteins used for analysis are presented in Dataset S1.

A total of 3231 proteins were identified and quantified in the roots of *M. acutistipula* from *canga* and rehabilitating minelands (Figure 1A and Dataset S1). Among them, 436 differentially abundant proteins were significant in plants from *canga* and 145 in plants from RM (Figure 1B and Dataset S1). All proteins with significant differential abundances were identified in samples from both environments. The PCA of the differentially abundant proteins showed the separation of samples from the different sampling sites (Figure 1C). The PCA result suggests that, even though these proteins were present in the samples from both sampling sites, the difference in abundance is influenced by the characteristics of the areas where the plants were sampled. *Canga* samples formed a closer group, while RM samples showed the greater distance from each other.

### 3.3. Gene Ontology Annotation

**Figure 2:**
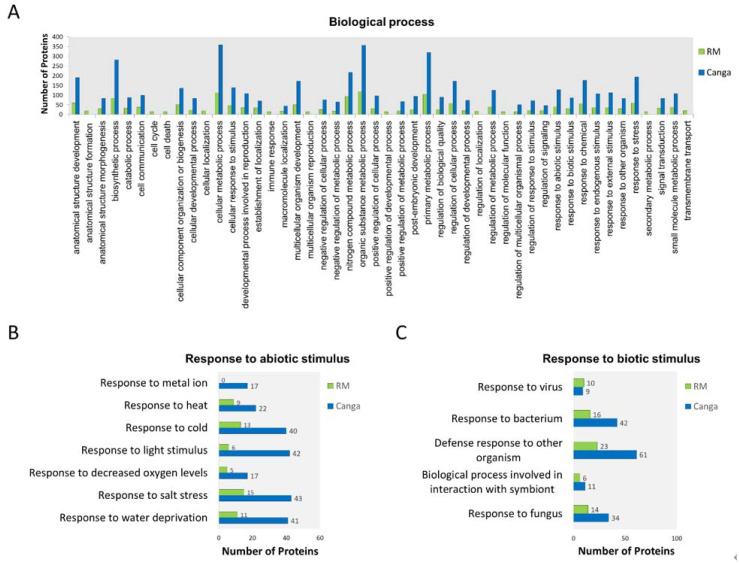
Gene Ontology (GO) annotation of differentially abundant proteins identified in *M. acutistipula* sampled from RM and *canga*. (**A**) GO annotation showing the GO terms in the category of biological process of more abundant proteins in plants from RM or *canga*. (**B**) Subgraph of the term response to abiotic stimulus. (**C**) Subgraph of the term response to biotic stimulus.

The most abundant proteins identified in the roots from the RM were assigned to 48 categories. Proteins from *canga* were related to 37 biological processes (Figure 2A), including proteins involved in responses to abiotic and biotic stimuli. Despite identifying exclusive categories in RM plants, the proteins attributed to these processes were also included in other terms common to plants from both environments. Therefore, they were not made up of exclusive proteins in RM plants. Among the categories to which the proteins involved in responses to abiotic stimuli were assigned, we highlight responses to water deprivation, salt stress, temperature (heat and cold), and to metal ions (Figure 2B). Proteins involved in responses to biotic stimuli have been attributed, among others, to responses to viruses, bacteria, and fungi, including biological processes involved in symbionts (Figure 2C).

**Figure 3:**
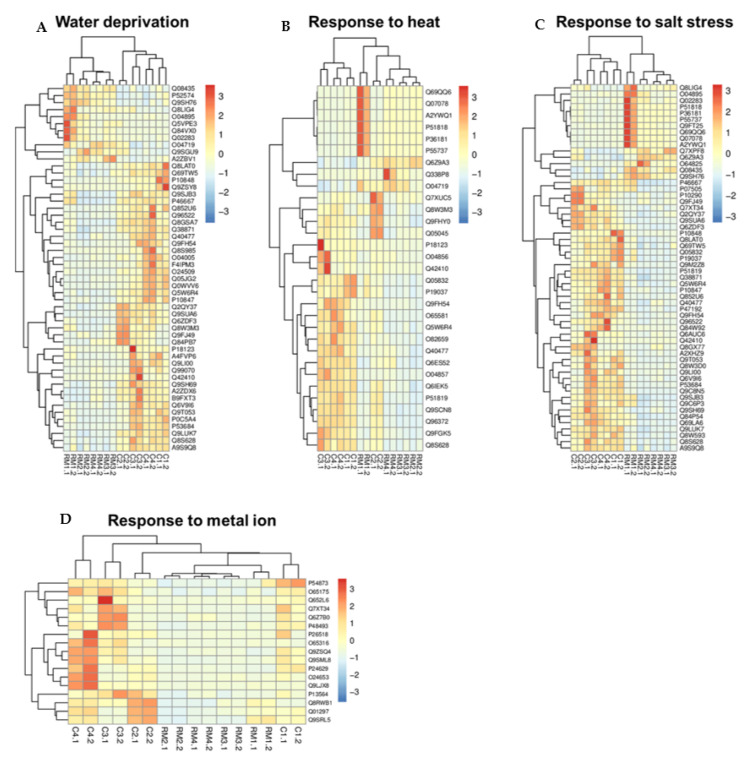
Hierarchical clustering of differentially abundant proteins related to abiotic stimulus in *M. acutistipula* sampled from RM (RM1.1, RM1.2, RM2.1, RM2.2, RM3.1, RM3.2, RM4.1, and RM4.2) and *canga* (C1.1, C1.2, C2.1, C2.2, C3.1, C3.2, C4.1, and C4.2). (**A**) Water deprivation. (**B**) Response to heat. (**C**) Response to salt stress. (**D**) Response to metal ion. The red and blue colors represent the highest and lowest intensity values, respectively. The accession numbers represent the proteins used for the analysis and can be viewed in Dataset S1.

**Figure 4:**
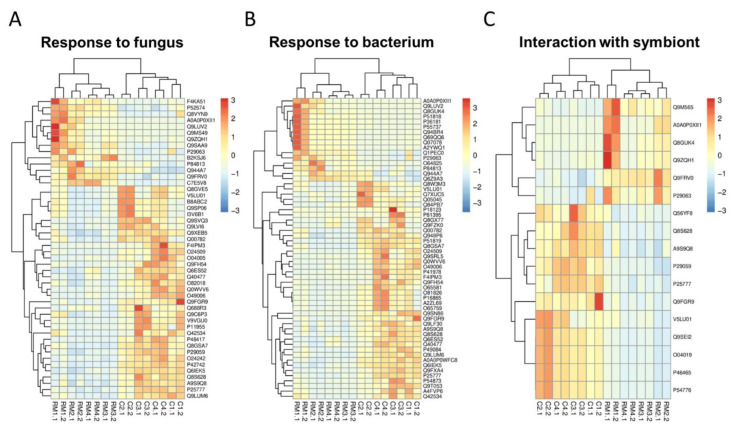
Hierarchical clustering of differentially abundant proteins related to biotic stimulus in *M. acutistipula* sampled from RM (RM1.1, RM1.2, RM2.1, RM2.2, RM3.1, RM3.2, RM4.1, and RM4.2) and *canga* (C1.1, C1.2, C2.1, C2.2, C3.1, C3.2, C4.1, and C4.2). (**A**) Response to fungus. (**B**) Response to bacterium. (**C**) Interaction with symbiont. The red and blue colors represent the highest and lowest intensity values, respectively. The accession numbers represent the proteins used for the analysis and can be viewed in Dataset S1.

Heatmaps were created with the proteins included in the most representative categories of response to abiotic (Figure 3) and biotic (Figure 4) stimuli, and also considering the characteristics of the environments. The hierarchical groupings separated the most abundant proteins in the roots of *canga* or RM plants into two well-defined groups, according to the patterns of intensities of the proteins identified in each one (vertical axis). Most replicates of RM and *canga* plant samples were also grouped separately by their similarity in protein intensity values (horizontal axis).

In the heatmaps of Figure 3 and Figure 4, it can be seen, therefore, that the highlighted proteins related to responses for water deficit (Figure 3A), heat (Figure 3B), salt stress (Figure 3C), and metal ion, as well as those attributed to responses to fungus (Figure 4A), bacterium (Figure 4B) and symbionts (Figure 4C), were identified in plants from both environments, being more abundant in *canga* plants. Response to metal ion (Figure 3D) represents the only group in which all proteins showed higher levels in *canga* plants.

## 4. Discussion

PCAs from the proteomes of *M. acutistipula* suggested that the differential abundance of proteins was related to the environments where the plants grew. *Canga* samples were more clustered, while distances between RM plant samples indicate a more significant heterogeneity of plants grown in these ecosystems. This same pattern was observed in a study of the proteome of *Dioclea apurensis* from plants growing in rehabilitating minelands [20]. However, there is still no knowledge about other factors, such as the differences in the genotypic variability of these populations between RM and *canga* plants, which are determinants in the observed patterns.

The proteins identified provide new evidence at the molecular level about adaptive mechanisms in native species in *canga* ecosystems. Differentially abundant proteins are highlighted here, especially those attributed to water deficit, heat, response to metal ions, and associations with soil microorganisms. These proteins are the most representative, especially in *canga* plants, indicating that this environment is severe. In addition to these proteins being involved in the good development of this species in stressful environments, the results also suggest a relationship with the effects of changes in the RM ecosystem, which interfere with the biological properties of the root system and the average growth of plants. These traits are advantages that favor the establishment of these species in RM.

A recent study has characterized the protein profile and the symbiotic interactions that allow the growth of *Dioclea apurensis* in post-mining areas, showing that proteins involved in the responses to abiotic stresses and associations with soil microorganisms are at the core of the metabolic modifications evolved in *canga* [20]. The same study observed that *D. apurensis* establishes non-specific interactions with soil microorganisms in *canga* and RM. Costa et al. [21] showed that *M. acutistipula* also sets non-specific interactions with soil microorganisms, including beneficial taxa such as nitrogen-fixing bacteria, mycorrhizal fungi, and other beneficial endophytes, both in *canga* and in RM. Such characteristics seem to be conserved between the species *M. acutistipula* and *D. apurensis,* both belonging to the Fabaceae family. Hence, molecular adaptations acquired in *canga* are transmitted to subsequent generations allowing a favorable gene regulation for tolerance to stressful environments [34,35,36]. Therefore, identifying these proteins in plants grown in RM can indicate the acquired adaptation of this species to stressful situations over the years.

### 4.1. Proteins Related to Abiotic Stimulus

A pool of enzymes related to water deprivation, temperature variations, salt stress, and response to metal ions was identified (Figure 2B and Figure 3), which are considered limiting factors to which native species need to adapt for their establishment in *canga* [3,16,19] or even in rehabilitating minelands [6,7,8,9]. These proteins were more abundant in *canga*. Among them are transcription factors, such as the sensitive to proton rhizotoxicity 1 (also known as STOP 1), a zinc finger protein that plays a critical role in stress tolerance [37] (Dataset S1). Additionally, there was a high level of the dehydration-responsive element-binding protein 2 transcriptional activator (Dataset S1). This family of transcription factors is known to confer tolerance to abiotic stresses such as salt, drought, and heat [38]. Although the highest levels of transcription factors occur in *canga* plants, the presence of these molecules in RM plants suggests the importance of the species’ adaptation in both environments due to possible environmental stresses.

Proteins involved in Ca^2+^ and abscisic acid (ABA) signaling are among the most representative of those attributed to abiotic stresses. These results help to understand the importance of incorporating Ca^2+^ and ABA signaling pathways for the adaptations of species such as *M. acutistipula* to stressful environments. These proteins were also identified in plants grown in both environments, with higher levels in *canga* plants.

Calcium-dependent protein kinases (CPKs) act in stimulus-specific recognition of environmental stresses, being the primary Ca^2+^ sensors that trigger responses to specific stimuli [39,40,41]. In our dataset, proteins involved in Ca^2+^ signaling were the most plentiful ones (Dataset S1 and Figure 3). CPK proteins participate in several abiotic stresses tolerance responses, including regulation of ABFs subfamily bZIP transcription factors [42], regulation of aquaporins and hydraulic conductivity of the roots [41,43], regulation of the abscisic acid-induced stomatal closure via S-type anionic channels (SLAC1) [39], regulation of ABA-responsive transcriptional factors, and ion channels [39,40], among others. Therefore, the higher levels of CPKs are a standard mechanism in plants growing under abiotic stress conditions and contribute to the establishment of *M. acutistipula* in the rehabilitating minelands by regulating several stress-responsive proteins.

Among the proteins involved in Ca^2+^-dependent signal decoding identified in this study are calmodulins (CaMs), CaM-like proteins, Calcineurin-like proteins (CBLs), and CBL-interacting protein kinases (CIPKs) [44,45,46]. Studies have shown that these proteins play an essential role in the biosynthesis and sensitivity of ABA in responses to osmotic stress [44,45]. These results highlight the importance of Ca^2+^ and ABA signaling in adapting *M. acutistipula* in *canga* and RM.

In our study, several proteins involved in the ABA pathway were identified, including ABA receptor pyrabactin resistance (PYL12), ABC transporter G family member 51, abscisic acid 8′-hydroxylase 1, and protein phosphatase 2C (PP2C), which were more abundant in *canga* plants (Dataset S1 and Figure 3). In RM plants, the abscisic stress-ripening protein 5 and PP2C 77 were more abundant (Dataset S1 and Figure 3). Under drought conditions, salt concentration increases, producing osmotic stress on plant cells. In response, plants synthesize ABA triggering a signaling cascade to induce stomatal closure and reduce water loss [47,48,49]. The main ABA signaling pathway includes key regulators of osmotic stress and ABA responses, including ABA receptor (PYR1/PYLs), PP2C, as well as SNF1-related protein kinase 2 (SnRK2) family proteins [50,51]. Similarly, ABC transporter G family member 51 is a member of the ATP-binding cassette (ABC) family associated with ABA transport in response to water deficit [48,52].

PP2C proteins mediate the ABA signaling pathway by negative feedback, dephosphorylating SnRK2s, preventing the phosphorylation necessary to activate ABRE-binding transcription factors and the transcription of ABA-responsive genes. PP2Cs also downregulates the activation of SLAC1, a vital ion channel in guard cells that regulates stomatal closure and controls water loss and CO_2_ supply under stress [53]. These proteins are essential for the stomatal opening and closing in response to environmental stresses [39,52], contributing the greater tolerance of plants to drought events reported mainly in *canga*. Furthermore, one of the primary catabolic pathways for controlling ABA content is triggered by the abscisic acid 8′-hydroxylase [54], which also was identified in this study (Dataset S1 and Figure 3).

*Canga* plants also showed higher levels of dehydrin RAB16C (Dataset S1 and Figure 3). Dehydrins are included in a group of late embryogenesis-abundant proteins involved in growth, development, and stress responses [35]. This protein was assigned to drought (Figure 3), agreeing with studies indicating that dehydrin genes are induced by dehydration stress in plants [35,55]. Other studies also point to the increased dehydrins in the salt stress response and osmotic change [35]. Additionally, the dehydrin network involves the ABA signal transduction pathways, including PYLs, PP2Cs, SnRKs, and BZIPs, and the Ca^2+^ signal transduction pathways, including the CaMs, CDPKs, CBLs, and CMLs [35]. This result suggests dehydrin as a critical protein in adapting *M. acutistipula* to drought events.

Ethylene response factors (ERFs) showed higher levels in *canga* plants. ERF4 and ERF114 were also assigned to drought and salt stress in *M. acutistipula*, while RAP2-9 was assigned to heat. This group of proteins regulates genes involved in biological processes such as growth, development, and responses to environmental stresses [56]. In addition, a subset of ERFs recognizes the dehydration-responsive element with a conserved sequence in stress-responsive genes to regulate responses to these stresses [38,56]. In agreement with these studies, a dehydration-responsive element-binding protein 2B was also identified with higher levels in *canga* plants (Dataset S1 and Figure 3).

Ethylene has an antagonistic interaction with ABA in response to abiotic stresses, including drought, heat, and high salinity [56]. This interaction is essential in regulating stomatal movement for balance during osmotic stress [56]. Ethylene and ABA also interact with auxin in different tissues and stages of plant development and response to environmental stresses [56,57]. In this study, the proteins auxin response factor 15 and auxin-responsive protein IAA27 were identified, showing higher levels in *canga* plants (Dataset S1 and Figure 3). These proteins are involved in auxin signaling pathways and are attributed to positive responses to environmental stresses such as drought [58,59], agreeing with what was shown by the GO analyses (Figure 2B and Figure 3).

Iron and other metal residues available in substrates can cause damage to plants established in *canga* or rehabilitating minelands [10,11,60,61]. One of the principal causes of excess metal toxicity is the displacement of essential metals in key biomolecules [62]. To survive in these environments, plants need to develop strategies to prevent root uptake and reduce long-distance transport of metal ions [63,64], suggesting that these are the main causes of all highlighted proteins with functions attributed to metal ion response that were more abundant in *canga* plants (Figure 3). Among them are heavy metal-associated isoprenylated plant protein 21 (HIPP) and ferritin 2 (FER), chloroplast. HIPPs and FER are among the most studied proteins for their roles in the homeostasis and detoxification of heavy metals in plants. HIPPs belong to a metal-binding metallochaperone group characterized by a heavy metal-associated domain and a C-terminal isoprenylation motif [65]. HIPPs are involved in tolerance to biotic stresses, such as defense against pathogen attack, and abiotic stresses, such as salt stress and water deficit [66]. However, efforts to characterize its functionalities mainly focus on its role in the homeostasis and detoxification of plants stressed by heavy metals [66,67]. They are found only in vascular plants, acting as critical proteins to safely transport metal ions within the cell to avoid harmful reactions [65,66,68]. Regarding FER, it represents one of the main proteins involved in free iron homeostasis in plants by carrying out the cytoplasmic sequestration of high amounts of soluble iron [69,70], transporting and accumulating excess iron that is safe and bioavailable in vacuoles, reducing the adverse effects of this metal in the intracellular environment.

### 4.2. Proteins Related to Biotic Stimulus

Several proteins related to interaction with microorganisms showed higher levels in *canga* plants. On the other hand, these proteins were also identified in the roots of *M. acutistipula* from RM (Figure 2C). Among them, the critical proteins with a reported role in biocontrol activity against phytopathogenic species include endochitinases [71], farnesene synthase [72], pectinesterase inhibitor [73], heat shock proteins [74], and chitin elicitor receptor [75] (Dataset S1 and Figure 3B). This is directly related to microbial diversity in rehabilitating areas after mining [21], modifying the microbes interacting with the plant roots, many of which can have a pathogenic lifestyle. This study also detected several proteins that have roles in establishing symbiosis (Figure 4). This group of proteins includes LysM receptor kinases [76], chitin receptor kinases [77], heat shock proteins [78], and subtilisin-like proteases [79]. The accumulation of these proteins underlines active symbiosis, which can support the establishment of *M. acutistipula* in RM. The symbiosis with beneficial microbial taxa has been described as essential to improving plant establishment in post-mining areas [21,80,81]. Symbiosis with arbuscular mycorrhizal fungi and nitrogen-fixing bacteria is the most crucial studied area in microbial-assisted phytoremediation in metalliferous soils such as Amazonian *cangas* [82,83]. Therefore, the accumulation of proteins involved in response to the infection of microorganisms and those involved in symbiosis is an additional mechanism that characterizes the growth of *M. acutistipula* in the RM.

## 5. Conclusions

This study showed that critical molecular mechanisms support the establishment of *M. acutistipula* in rehabilitating minelands over time. These mechanisms acquired in the severe *canga* ecosystem explain why *M. acutistipula* is one of the most promising native species used in mineland rehabilitation programs in the eastern Amazon. Further studies must evaluate the presence of specific molecular traits that can help to understand the survival of native species used in mineland rehabilitation programs.

## Figures and Tables

**Table 1:** Characteristics of substrates associated with *Mimosa acutistipula* growing in *canga* and rehabilitating minelands (RM). Soil results are mean ± standard deviation for n = 4.

	RM	*Canga*
Organic carbon (g kg^−1^)	8.2 ± 2.9	44.9 ± 5.7
Organic matter (g kg^−1^)	14.3 ± 5.0	77.4 ± 9.8
pH (H_2_O)	6.0 ± 0.17	4.65 ± 0.60
Available P (mg dm^−3^)	15.65 ± 3.55	1.0 ± 0.96
Fe (mg dm^−3^)	11.25 ± 4.19	372.5 ± 92
Clay (g kg^−1^)	488.7 ± 37.5	287.5 ± 103
Sand (g kg^−1^)	355 ± 64.5	625 ± 177
Silt (g kg^−1^)	162 ± 32.2	87.5 ± 75

## Data Availability

The proteomic data have been deposited in the massive repository under the accession MSV000090601.

## Supplementary Materials

The following supporting information can be downloaded at: https://www.mdpi.com/article/10.3390/ijerph192114441/s1, Dataset S1: Protein report of *Mimosa acutistipula* in *canga* and RM plants.
